# Personality traits and occupational status-evidence from China

**DOI:** 10.1371/journal.pone.0284050

**Published:** 2023-04-07

**Authors:** Yanrong Liu

**Affiliations:** School of Economics and Management, University of Science and Technology Beijing, Beijing, China; University of Milano–Bicocca: Universita degli Studi di Milano-Bicocca, ITALY

## Abstract

This paper estimates the relationship between personality traits measured by the “Big Five Model” and occupational status with a nationally representative household survey from China. I find that four of the five personality traits except extraversion are significantly associated with occupational status in terms of occupational choices, occupational prestige, and socioeconomic status. In particular, conscientiousness is the most important predictor among the five dimensions of personality traits. The findings also suggest that the returns of personality traits to occupational status are higher for females.

## Introduction

In recent years, personality traits such as communication ability, persistence, motivation, and self-esteem are playing an increasingly important role in explaining socioeconomic outcomes [1, 2]. A growing literature has shown that personality traits are associated with one’s economic behaviors and labor market outcomes in developed countries [1, 3–5]. Few studies, however, have examined the role of personality traits in the labor market in developing countries [6–9].

Compared with developed countries, the returns of personality traits may differ due to different labor market situations and cultures in developing countries [10, 11]. For instance, most people work in informal sectors and lack social protection in the developing labor markets, where social networks are employed frequently in job hunting and the returns of social skills may be larger [12]. In addition, some specific personality traits may be valued in some countries but not in others due to cultural differences. For example, US culture is highly individualistic [13]. In contrast, Chinese culture is strongly influenced by Confucianism and is more relational [14], which emphasizes sociability and cooperation in China’s labor market. Thus, the evidence on personality traits from developed countries may not hold in the setting of developing countries, where the research on personality traits is scarce due to the lack of data and the difficulties in measuring personality traits.

Most of the literature has focused on the direct effect of personality traits on labor market performance, such as income or earnings [1, 4, 6]. Although many scholars have pointed out that the potential mechanism through which personality traits may influence economic outcomes is occupational choices and career promotion, little literature has conducted empirical research by incorporating occupational choice into a framework of personality traits returns, especially in developing countries [6]. In addition, occupational segregation has implications for other labor market outcomes. The gender wage gap often results from gender segregation across industries or occupations [15]. Thus exploring the relationship between personality traits and occupational attainment could provide an explanation for occupational segregation and a better understanding for gender wage gap in China’s labor market^φ^.

The paper examines the association between personality traits and occupational status based on the Chinese Family Panel Studies (CFPS), a nationally household representative survey in China. The CFPS data contains rich information on personality traits and direct occupational status indicators. The paper measures one’s personality traits with Big Five Model (Conscientiousness, Extraversion, Agreeableness, Openness, and Neuroticism), which were introduced by McCrae and John [16] and are most commonly used in the recent psychology and economics literature [7, 17, 18].

The main results show that four of the five personality traits except extraversion are significantly associated with one’s occupational status in terms of occupational prestige, socioeconomic status, and occupational choices. In particular, conscientiousness is the most important and stable predictor for occupational status. For instance, conscientiousness significantly increases one’s socioeconomic status and occupational prestige scale by 5.65% and 1.73%. An increase in conscientiousness by one standard deviation is also associated with a 5.16% higher likelihood of being managers or professionals. I also find that the returns of personality traits to occupational status are higher for females.

The contributions of the paper are as follows. First, while the literature almost focuses on the role of personality traits in occupational status across developed countries, I explore the issue in China with a nationally representative survey, the largest developing country in the world. Second, this paper is among the few studies suggesting that the returns of personality traits to occupational status are higher for females, which indicates that raising non-cognitive skills is an important way to improve women’s occupational status and reduce gender occupational segregation in China’s labor market.

## Theoretical framework and literature review

### Personality traits

Personality traits are playing an important role in explaining economic behaviors and have been proven to be partially inherited and relatively stable across the adult life span [19]. With attention to personality traits, psychologists have gradually developed various measurement tools to measure personality traits. The Big Five Model (Open, Conscientiousness, Extroversion, Agreeableness, and Neuroticism) proposed by McCrae and John summarizes the variety of facets of psychological traits as the five independent categories [2, 16]. These factors represent personality at the broadest level, and each factor includes a large number of distinct and specific personality characteristics [20]. Most variables used to measure personality in the research are conceptually and empirically related to the Big Five model [16]. For example, “Conscientiousness” is related to perseverance and self-control; “Extraversion” includes many aspects of interpersonal skills; and “Neuroticism” is associated with locus of control and self-perception. The Big Five model has been proven to be validated across different languages and cultures and is widely accepted in personality and social psychology literature [7, 17, 18].

Besides the Big Five Model, there are several other measures of personality traits, such as locus of control [21], self-esteem [22], and the Big Three [23]. Locus of control is an important type of non-cognitive ability, referring to a person’s general beliefs about the potential determinants of events in life [21]. A person with an internal locus of control believes that life events are usually caused by their actions or characteristics, such as personal efforts and education. In contrast, a person with an external locus of control believes that their life is determined by fate, luck, or other external forces. Self-esteem refers to an individual’s sense of his value, or the extent to which a person values or appreciates himself, and is usually measured by the Rosenberg Self-esteem scale. Eysenck also offered the Eysenck Personality Questionnaire (EPQ) to briefly measure personality traits with just three major dimensions of personality (Extraversion, Neuroticism, and Toughmindedness) [23]. In addition, Digman proposed a more parsimonious model in which the Big Five factors are reduced further to two higher-order factors. One is principally related to dimensions of Agreeableness, Conscientiousness, and Emotional Stability, and the other meta-traits include the dimensions of Extraversion and Intellect [24].

### Theoretical framework

The human capital model studies the relationship between human behavior and human capital formation and occupational outcomes [6, 25]. With the study of personality traits in psychology, the literature began to incorporate personality traits into the model to explain individual economic behaviors [1]. In the labor market, occupation types are assigned to individuals through the interactions between the demand side factors for various labor services by employers and the individual’s supply decisions [26]. On the one hand, different occupations and positions have specific requirements for personality traits, with some traits valued in certain occupations but not in others. On the other hand, individuals differ in their productive skills and preference for various utilities related to labor supply, leading to differences in worker’s labor outcomes [26, 27]. Assuming that individuals choose occupations to achieve desired career development and obtain the highest rewards, each person will make an optimal investment in his human capital [26]. Therefore, individuals who are constrained by firm profit maximization will choose occupations that they are better at and have a comparative advantage to maximize their utility [10, 26, 27]. An individual’s occupational attainment can be described as:

Max(Occu_attainment)=f(S,I,H,X)+ε (1)

Human capital variables include individual skills (S), other individual characteristics (I), household socioeconomic characteristics (H), and other factors (X) that affect labor market outcomes [25].

Individual skills include cognitive skills and personality traits. Cognitive skills are often referred to as test scores or literacy and numeracy tests, which are strongly correlated with an individual’s career achievement. Personality traits skills such as motivation, persistence, and self-esteem play an increasingly important role in one’s occupational attainment. People with high level of non-cognitive abilities are more likely to look for more challenging jobs and thus have higher occupational status.

As the human capital theory suggests, other individual characteristics (I) such as age, marital status, education level, and health status are important predictors in determining their productivity in various occupation types and occupational attainment [28]. In addition to individual characteristics, family background also matters for the formation of human capital and thus labor market outcomes (H). Finally, other factors (X) such as technology, general labor market situation, and economic environment is also associated with individual decision-making in the labor market [29]. The analysis includes measures for each facet of human capital described above.

Different personality traits have different effects on individual labor market performance [27]. Conscientiousness depicts how an individual handles tasks and refers to the tendency to be self-controlled, organized, responsible, and hardworking. Previous study suggests that conscientiousness has a generally positive effect on an individual’s career success and labor market performance [6, 26, 30]. Conscientiousness is also the most consistent personality trait related to performance across all occupation groups. Extraversion is an orientation of one’s interests and energies toward social interaction. It includes assertiveness, talkativeness, sociability and is valued in social jobs. People with high level of sociability are more likely to choose jobs that involve more interpersonal interactions [20]. Agreeableness refers to the individual’s interpersonal relationships and is associated with being friendly, cooperative, and sympathetic. In work, especially in teamwork, agreeableness helps individuals finish work better and is related to an outstanding career. Openness captures how individuals value new experiences and changes and the tendency to be open to new ideas and unconventional values. The trait is associated with an active imagination, intellectual curiosity, and desire for new experiences and ideas. Openness to experience is essential for people engaged in creative business or jobs requiring innovation. Neuroticism captures how an individual behaves under stressful situations and includes traits such as irritability and anxiety. People with emotional instability are less able to cope with stress and intense negative emotions always lead to lower productivity, thus lower occupational attainment [26, 31, 32].

The ways that individuals’ personality traits are matched to occupations is likely to differ between men and women. Previous studies have shown that gender differences in risk preferences, competitiveness, and achievement orientation [33]. Women have more feminine personality traits and more risk averse than men on average [34], thus having different preferences over the occupation types. Specifically, women are more likely to occupy safer jobs or lower-income-risk jobs [35]. In addition, women also have higher levels of agreeableness than men, which increases the tendency to choose low-paid service occupations [36].

### Literature review

Personality traits are considered at least as important as cognitive abilities [1, 37] and have critical impacts on a variety of life outcomes, including academic outcomes [14, 20], health status [38], and social behaviors, such as divorce and crime activities [1, 39]. Heckman, Stixrud and Urzua [1] examined the relationship between personality traits measured with loss of control and self-esteem and a variety of life outcomes based on the National Longitudinal Survey of Youth. The results showed that the non-cognitive test score is significantly and positively associated with the probability of being a four-year college graduate. Non-cognitive skills are also associated with the likelihood of daily smoking for men and participating in illegal activities.

A wide range of studies also examined the association between personality traits and labor market outcomes in developed countries, including entrepreneurial choices [40], occupational choices and status [16, 26], job performance [32], employment and earnings [3–5, 31, 41]. For instance, with a large subsample of siblings and sibling fixed effects, Fletcher showed that personality traits measured by the Big Five model has important associations with employment and wages in adulthood [31]. Blázquez and his colleagues [37] examined the relationship between non-cognitive skills and occupational status and earnings among European university graduates and suggested that leadership is the most related competence to managerial positions for men, while initiative and enterprise are the most relevant in managerial positions among women. Ham, Junankar and Wells [26] showed that conscientiousness significantly increases the probability of attaining a white-collar occupation.

Few studies, however, have examined the role of personality traits in the labor market in developing countries [6–8]. For instance, Bühler, Sharma and Stein [6] investigated the role of non-cognitive skills in occupational attainment and earnings using micro-level data from rural Thailand and Vietnam. The results showed that higher levels of conscientiousness increased the probability of choosing to work as a craft worker or laborer or to be self-employed, while lower levels of neuroticism were associated with higher earnings. Based on data from Indonesia, Adhitya, Mulyaningsih and Samudro [7] suggested that extraversion personality is a strong predictor of workers’ performance, particularly for upper-income groups.

In the setting of China, where the analyses are based on, the research on non-cognitive skills is limited and mostly focused on educational attainment [42], stock investment [15], entrepreneurial choices [43, 44], and employment and earnings [9, 45, 46]. For instance, with data from the China Employer-Employee Survey (CEES), Cheng and Li [9] suggested that openness and conscientiousness personalities play a significant role in promoting workers’ wages.

To sum up, the research on occupational attainment returns to personality traits has largely focused on developed countries, such as the U.S. and European countries. While there is a growing interest in this issue in developing countries, the literature mainly focuses on other labor market outcomes such as entrepreneurial choices and earnings. Occupational status may reflect an individual’s income, education, and social standing. This paper aims to fill the gap by estimating the relationship between personality traits and occupational status in terms of occupational prestige, socioeconomic status, and occupational choices based on a nationally representative household survey in China.

## Methodology

### Data and measures

The main dataset used in this paper is the 2010 and 2012 wave of China Family Panel Studies (CFPS), which is a nationally representative survey of Chinese communities, families, and individuals, covering 25 provinces/regions (municipalities and autonomous regions). The survey design is based on similar surveys in developed countries, and most of the questions are covered in the four U.S. counterpart datasets (PSID, CDS, HRS, and NLSY). The sample in 2010 was chosen from 16 counties from each of the five large provinces (Shanghai, Liaoning, Henan, Gansu, and Guangdong) and 80 counties from 20 other provinces, then chose 2 or 4 communities in each county, finally 28–43 households were sampled from each village or resident committee. It contains 14,960 households and 42,590 individuals. The CFPS data collects rich information on demographic and socioeconomic characteristics, economic activities, education outcomes, family dynamics, and relationships [47].

The 2010 and 2012 CFPS questionnaires collect the five factors of personality straits, and each of these traits contains several specific facets concerning the Big Five Personality Factors. Respondents were asked how much they agreed with different statements related to personality traits. Most personality traits items are based on questions in the 2010 CFPS questionnaire, except the item of “trust” of agreeableness with the corresponding question in the 2012 CFPS survey. I adjust the score range of all items for each trait to 1–5, with 1 being strongly disagreed and 5 totally agreed. I average the item scores for each trait and use it as an indicator of the strength of individual personality traits. Table A in S1 File provides a list of 13 items and their corresponding survey questions in the CFPS questionnaire.

The main outcome variables in the paper are one’s occupational status. The CFPS survey converts the occupational codes Chinese Standard Classification of Occupations (CSCO) to International Standard Classification of Occupation codes (ISCO-88), and constructs three indicators to measure one’s occupational socioeconomic status: International Socio-Economic Index of Occupational Status (ISEI), Treiman’s Standard International Occupational Prestige Scale (Treiman’s SIOPS), and EGP occupational types (Erikson and Goldthorpe’s Class Categories, EGP). Occupational prestige scores are calculated based on Treiman’s Standard International Occupational Prestige Scale (Treiman’s SIOPS) by Treiman [48] and range from 13 to 78, which indicates a level of power and privilege for individuals according to their occupations across modern societies and social groups. ISEI scores are calculated based on the average education level and income of each occupation and range from 19 to 90, indicating the socioeconomic status of different occupations. Treiman’s SIOPS and ISEI are continuous variables, with higher scores indicating higher occupational socioeconomic status.

The EGP categorizes occupations into ten types according to employment status and skill levels of each occupation in the CFPS survey. I define five occupation types in the analysis: self-employed (self-employed with and without employees), managers or professionals (higher controllers and lower controllers), routine non-manual workers, manual workers (manual supervisors, skilled manual workers, semi-unskilled manual workers), and agricultural workers (agricultural laborers and self-employed agricultural workers).

Control variables include individual-level, household-level, and community-level characteristics. The respondent’s age is a continuous variable ranging from 16 to 60. Male dummy variable equals to one if the respondent is male and zero otherwise. Married is an indicator equal to a value of one if the respondent reported being married currently and zero otherwise. Based on the ethnic identification, I generate a dummy variable “ethnic minority” taking on a value of one if the respondent is identified as not an ethnic Han. Education level is one’s highest education level and is categorized into three levels: primary school, middle school, and high school or above. In China, the household registration system divides individuals into different social levels [49]. I construct a dummy variable “urban hukou”, which equals one if the respondent possesses non-agricultural household registration. Based on the answer to health status, I generate a “self-rated health status” variable on a 5-point scale, with a higher value indicating better health status. I also add family size in the regression model measured by the number of siblings. The analysis also accounts for an indicator variable for whether the father or mother indicates his or her political status as a member of the Communist Party of China (CCP). Urban community is an indicator variable equal to one if the respondent lives in the urban areas now and zero otherwise.

To examine the relationship between non-cognitive skills and one’s occupational status, I restrict the analysis to samples aged 16 to 60. Finally, I get a sample of 12451 cases for the main analysis. In addition, to examine the relationship between personality traits and one’s occupational types, the analysis keeps the samples engaged in non-agricultural work and gets 4925 cases.

### Empirical model

The study estimates the role of personality traits in occupational types using a multinomial logit model. The probability of each occupation is estimated by:

$$Pr(Occu_{i}=o|{\mathrm{Noncog}}_{i},X_{i})=\frac{\exp(\beta_{1}{\mathrm{Noncog}}_{i}^{\prime }+X_{i}^{\prime })}{\sum_{o=1}^{4}\exp(\beta_{1}{\mathrm{Noncog}}_{i}^{\prime }+X_{i}^{\prime })}+\varepsilon_{i},i=1,\ldots n;o=1,\ldots 4$$ (2)

I then adopt an OLS approach to estimate the association between personality traits and occupational prestige and socioeconomic status with the following form:

$$Occu\_status_{i}=\beta_{0}+\beta_{1}Noncog_{i}+\beta_{2}X_{i}+\varepsilon_{i}$$ (3)

I define a categorical dependent variable *Occu* in Eq (2) that takes the values 1, 2, 3, or 4 depending on if the respondent *i* is self-employed, is a manager or professional, is employed as a non-routine manual worker, or a manual worker. The analysis takes the last category as the reference group. *Occu_status* in Eq (3) is one’s occupational prestige and socioeconomic status measured by SIOPS and ISEI scores. The coefficient of interest is *β*_1_ on the key explanatory variable *Noncog*, including consciousness, extraversion, openness, agreeableness, and neuroticism.

The vector X in Eqs (2) and (3) contains the individual-level (one’s age, gender, marital status, ethnic minority, health status, education level), household-level (parents’ education level, political status, number of siblings), and community-level variables (urban community). As the human capital theory suggests, education level, experience, health status, an individual’s innate ability are important predictors in explaining their productivity in the labor market [28]. Health is a form of human capital that increases individual productivity by improving workforce quality. Education level is a powerful predictor of occupational attainment, which determines their occupational status when entering the labor market and their chances of subsequent mobility and promotion. People with high education levels are more likely to obtain favorable conditions in career development and get decent work with high social prestige, such as professional technicians and managers, thus positively promoting occupational status [50]. In addition, with the restrictions of the household registration system in China, individuals are divided into different social levels. Urban residents usually have access to better educational resources and job opportunities [49].

In addition to individual characteristics, previous studies have suggested that family socioeconomic status may affect the individual’s human capital investments and occupational outcomes [31, 50]. Compared with disadvantaged families, children who grow up in wealthy families have a higher level of human capital investment in their early life to shape personality traits related to occupational attainment. Parents’ education level may affect children’s occupational status through children’s education [50] or preferences [51]. Generally, People with poorly educated parents are more sensitive to economic incentives and risk-averse in their occupational choices [51]. Given the complete dominance of the Communist party in China’s political system, party membership signals an important political affiliation. Therefore, parents’ party membership could increase the probability of being a party member for the individual and generate political and social capital in the labor market [52]. Therefore, I add family characteristics in the regression model. In addition, with well-developed capital and labor markets in urban areas, urban residents have access to resources that are beneficial to career development. Thus, the regression model also accounts for community characteristics.

The estimation of the above equation may be biased due to endogeneity concerns of personality traits [6, 31]. Some unobservable factors may be correlated with both personality traits development and occupational status, such as genetic endowments and family background. For example, individual personality characteristics are shaped by different innate abilities or genetic endowments that affect one’s occupational choices accordingly [31, 46]. In addition, parental occupational choices and income are also related to the accumulation of non-cognitive skills and one’s occupational status through intergenerational transmission. In these cases, the results may overestimate the correlation between personality traits and occupational socioeconomic status.

In addition to possible omitted variable bias, the estimates may be subject to measure errors and reverse causality concerns because the available measures of personality traits are mostly based on self-reported [45, 53]. Since the personality traits are measured by a set of questions, measurement errors may exist when respondents make errors or the question itself cannot fully capture the underlying concept [45].

While previous research suggests that an individual’s personality is partially inherited and relatively stable with adults over their lifetime [16, 19], it is possible that one’s job environments are related to his current levels of non-cognitive skills [6, 37, 53]. On the one hand, people with higher skills tend to have higher productivity and are more likely to occupy high-skilled jobs. On the other hand, personality traits may also be affected by success or failure at work. For instance, people with poor job performance may be likely to report a high neuroticism score or develop certain personalities related to the job to improve performance [24, 37].

However, it is difficult to find an exogenous source of variation in individual’s personality traits that unrelated to one’s occupational outcomes. To alleviate the endogeneity concerns, I first add a full set of cohort dummies to account for the non-linear effects of personality traits across cohorts. I continue to control for county fixed effects in the regression model to eliminate possible time-invariant unobservable confounding factors at the county level, such as local employment situations and economic development level. To further address possible omitted variables bias, I control for cognitive abilities measured by word test and math test scores in robustness checks. I also limit the sample to those aged 30 to 60 and repeat the analysis to eliminate possible reverse causality concerns.

In addition, self-selection into occupation types and occupational status could induce selection bias into the results, thus the samples are unlikely to be random. To avoid the sample selection bias in the analysis, I use the two-step Heckman model first proposed by Heckman in robustness checks [54].

## Results

### Descriptive analysis

Table 1 provides an overview of the share of respondents in each occupation, with agriculture employing the highest proportions and the lowest socioeconomic status. Approximately 21.12% of respondents report manual workers as their main occupation in the analysis. About 13.63% of respondents are professionals or managers with the highest prestige scale and socioeconomic status, and only a small proportion of respondents are self-employed.

**Table 1:** Overview of occupation types.

Occupation type	Frequency	Percentage (%)	Prestige scale	Socioeconomic status
Self-employed	915	9.68	33.646	36.219
Managers or professionals	1288	13.63	58.820	62.314
Routine non-manual workers	726	7.68	36.415	43.131
Manual workers	1996	21.12	33.041	32.059
Agricultural workers	4524	47.88	39.768	23.054

*Sources*: CFPS 2010 survey data.

Table 2 provides detailed descriptive statistics of personality traits, individual- and household-level characteristics for full samples. The results for females and males shown in Table B in S1 File suggest that males are more likely to be enterprising, gregarious, positive, trusting, and emotionally stable, therefore more likely to be managers or professionals and have higher occupational socioeconomic status. In addition, the individual and household characteristics differ significantly between females and males.

**Table 2:** Descriptive statistics.

	Full sample				
	N	Mean	SD	Min	Max
**Panel A: Occupational status**					
Occupational socioeconomic status score	12451	31.356	13.674	19	88
Occupational prestige score	12451	39.789	9.244	13	78
**Panel B: Big Five Model**					
Conscientiousness	12451	3.459	0.537	1	5
Extraversion	12451	3.958	0.664	1	5
Agreeableness	12451	3.833	0.684	1	5
Openness	12451	1.972	1.133	1	5
Neuroticism	12451	1.492	0.615	1	5
**Panel C: Individual characteristics**					
Age	12451	41.269	10.640	17	60
Male (%)	12451	53.001	0.499	0	1
Married (%)	12451	89.591	0.305	0	1
Ethnic minority (%)	12451	8.360	0.277	0	1
*Education level*					
Primary school (%)	12451	45.265	49.777	0	1
Middle school (%)	12451	32.606	46.877	0	1
High school or above (%)	12451	22.135	41.517	0	1
Self-rated health status	12451	4.287	0.930	1	5
Urban hukou (%)	12451	22.625	41.842	0	1
**Panel D: Household characteristics**					
Number of siblings	12451	2.991	1.873	0	14
*Father’s education level*					
Primary school (%)	12451	75.472	43.027	0	1
Middle school (%)	12451	15.083	35.790	0	1
High school or above (%)	12451	9.445	29.247	0	1
*Mother’s education level*					
Primary school (%)	12451	88.226	32.231	0	1
Middle school (%)	12451	8.216	2.746	0	1
High school or above (%)	12451	3.558	18.525	0	1
Father is party member	12451	15.830	36.504	0	1
Mother is party member	12451	2.273	14.904	0	1
Urban community (%)	12451	38.921	48.759	0	1

*Sources*: CFPS 2010 and 2012 survey data.

### Personality traits and occupational status

Tables 3 and 4 show the estimation results of the relationship between personality traits and occupational attainment in terms of occupational types, prestige scale, and socioeconomic status. I estimate a multinomial logit model where I use manual workers as the base outcome in Table 3 and report the marginal effects of each personality trait by occupation types in the results. Table 4 shows the results of occupational prestige and socioeconomic status with OLS regression. The results suggest that four of the five personality traits except extraversion are significantly associated with one’s occupational status^κ^.

**Table 3:** Personality traits and occupational types.

	(1)	(2)	(3)
	Self-employed	Managers or professionals	Routine non-manual workers
Conscientiousness	0.226**	0.480***	0.241**
	(0.111)	(0.108)	(0.122)
Extraversion	-0.057	0.069	-0.017
	(0.093)	(0.093)	(0.104)
Agreeableness	0.132*	0.260***	0.098
	(0.079)	(0.078)	(0.089)
Openness	0.005	0.081**	0.088**
	(0.042)	(0.038)	(0.043)
Neuroticism	-0.028	-0.109	-0.090
	(0.090)	(0.089)	(0.101)
Observations	4925	4925	4925

*Notes*: All regressions control for cohort dummies, gender, marital status, minority, education level, health status, number of siblings, parents’ education level, whether parents are party members, urban dummies, and county fixed effects. Robust standard errors clustered at the county level are shown in parentheses.

*** significant at 1 percent level

** significant at 5 percent level.

**Table 4:** Personality traits and occupational prestige and socioeconomic status.

	Prestige scale	Socioeconomic status
Conscientiousness	0.689**	1.772***
	(0.267)	(0.318)
Extraversion	-0.012	-0.061
	(0.176)	(0.194)
Agreeableness	0.463***	0.554***
	(0.123)	(0.174)
Openness	0.164*	0.287***
	(0.083)	(0.109)
Neuroticism	-0.301**	-0.158
	(0.128)	(0.159)
R^2^	0.151	0.439
Observations	12451	12451

*Notes*: All regressions control for cohort dummies, gender, marital status, minority, education level, health status, number of siblings, parents’ education level, whether parents are party members, urban dummies, and county fixed effects. Robust standard errors clustered at the county level are shown in parentheses.

*** significant at 1 percent level

** significant at 5 percent level

* significant at 10 percent level.

Conscientiousness, such as organization and achievement striving, is positively and significantly associated with one’s occupational status. Table 3 shows that conscientiousness is an important determinant for all occupations. For instance, an increase in conscientiousness by one standard deviation is associated with a 5.16% higher likelihood of being managers or professionals and a 0.90% higher likelihood of being self-employed. The results in Table 4 suggest that conscientiousness significantly increases one’s socioeconomic status and occupational prestige scale by 5.65% and 1.73%. Being conscientious contributes to individuals’ capability to learn, and persons displaying high levels of conscientiousness are always responsible, efficient, and hardworking in the labor market [6]. The findings are consistent with the previous literature in economics and psychology, indicating that conscientiousness is the most important and decisive predictor for occupational performance in developing and industrialized countries [6, 21, 30].

The agreeableness trait has a significant and positive correlation with one’s occupational status. In particular, being agreeable significantly increases prestige scale and socioeconomic status by 1.16% and 1.77% and significantly at 1% level. An increase in agreeableness by one standard deviation also relates to 2.92% higher likelihood of being managers or professionals. As the literature indicates, an individual with agreeableness traits is capable of working well due to his interpersonal skills and team cooperation spirit [30].

The openness results suggest that people scoring higher on this factor have a higher probability of choosing to be managers or professionals, or engaging in routine non-manual work. In addition, being open also significantly increase socioeconomic status by 0.92%. An open person, who is willing to entertain new ideas and unconventional values, is creative and enthusiastic in the workplace [55]. Individuals with high levels of openness usually prefer professional positions that require analytical and creative thinking [6], therefore getting higher occupational socioeconomic status.

Neuroticism shows how negative emotions affect individuals’ labor market performance. As predicted by literature, individuals who experience negative feelings more intensely might be less productive due to their unstable emotions or distress [21]. Being neurotic, such as being depressed and vulnerable, is significantly and negatively associated with one’s occupational prestige scale: it reduces one’s prestige scale by 0.76%. While the evidence suggests that more neurotic individuals are more likely to be professionals or managers [6], there are no associations between neuroticism and all occupation types in the analysis.

In addition, I do not find a significant association between extraversion and occupational status. The findings resonate with Adhitya, Mulyaningsih and Samudro [7], who used data from Indonesia and suggested that extraversion personality is a strong predictor of worker’s performance, particularly for upper-income groups.

### Robustness checks

Although I have controlled cohort and county fixed effects and a rich set of individual and household characteristics in the analysis, some factors may still confound the relationship between personality traits and occupational status. In this section, I do robustness checks to account for such concerns, and show robustness to alternative samples and measures of occupational status.

First, I repeat the analysis by including cognitive ability in the regression model. Cognitive skills play an important role both in one’s non-cognitive skills formation and one’s occupational status, however, non-cognitive skills could also affect cognitive development in turn. I add one’s cognitive ability measured by math and word test scores, and the results in Table 5 show that conscientiousness, agreeableness, openness, and neuroticism coincide with the main findings after controlling for one’s cognitive ability.

**Table 5:** Robustness checks.

	Occupational types					
	(1)	(2)		(3)		
	Self-employed	Managers or professionals		Routine non-manual workers	Prestige scale	Socioeconomic status
**Panel A: including cognitive skills**						
Conscientiousness	0.217*	0.468***	0.233*		0.651**	1.684***
	(0.111)	(0.109)	(0.122)		(0.266)	(0.309)
Extraversion	-0.059	0.017	-0.038		-0.036	-0.110
	(0.093)	(0.095)	(0.105)		(0.176)	(0.193)
Agreeableness	0.126	0.215***	0.069		0.425***	0.464***
	(0.079)	(0.079)	(0.090)		(0.121)	(0.173)
Openness	-0.001	0.052*	0.072*		0.138	0.228**
	(0.042)	(0.039)	(0.044)		(0.084)	(0.108)
Neuroticism	-0.030	-0.104	-0.089		-0.292**	-0.134
	(0.090)	(0.090)	(0.101)		(0.128)	(0.157)
Observations	4924	4924	4924		12450	12450
**Panel B: two-step Heckman model**						
Conscientiousness	0.226**	0.480***	0.241**		0.920***	2.129***
	(0.111)	(0.108)	(0.122)		(0.339)	(0.379)
Extraversion	-0.057	0.066	-0.020		-0.047	0.072
	(0.093)	(0.093)	(0.104)		(0.212)	(0.241)
Agreeableness	0.135*	0.260***	0.098		0.565***	0.734***
	(0.079)	(0.078)	(0.089)		(0.155)	(0.204)
Openness	0.004	0.081**	0.088**		0.194*	0.378***
	(0.042)	(0.038)	(0.043)		(0.107)	(0.134)
Neuroticism	-0.035	-0.108	-0.088		-0.375**	-0.098
	(0.090)	(0.090)	(0.101)		(0.168)	(0.229)
Observations	4925	4925	4925		9577	9577
**Panel C: samples aged 30–60**						
Conscientiousness	0.265**	0.467***	0.277*		0.876***	1.937***
	(0.127)	(0.125)	(0.149)		(0.297)	(0.347)
Extraversion	-0.066	0.098	0.054		0.005	-0.152
	(0.106)	(0.107)	(0.128)		(0.188)	(0.216)
Agreeableness	0.091	0.248***	-0.045		0.410***	0.525***
	(0.089)	(0.091)	(0.109)		(0.139)	(0.178)
Openness	0.000	0.085**	0.108**		0.192**	0.290**
	(0.047)	(0.043)	(0.051)		(0.086)	(0.113)
Neuroticism	-0.074	-0.051	-0.081		-0.212	-0.126
	(0.104)	(0.101)	(0.121)		(0.135)	(0.151)
Observations	3736	3736	3736		10358	10358
**Panel D: CFPS 2016 wave**						
Conscientiousness	0.135	0.349***	0.171		0.779***	1.377***
	(0.126)	(0.111)	(0.122)		(0.282)	(0.317)
Extraversion	0.045	-0.053	0.032		-0.205	-0.018
	(0.097)	(0.073)	(0.083)		(0.190)	(0.209)
Agreeableness	0.229***	0.156**	0.045		0.364**	0.303
	(0.084)	(0.066)	(0.086)		(0.157)	(0.186)
Openness	0.025	0.056*	0.079**		0.025	0.374***
	(0.040)	(0.034)	(0.039)		(0.096)	(0.106)
Neuroticism	-0.066	-0.117	-0.085		-0.140	0.110
	(0.097)	(0.083)	(0.094)		(0.148)	(0.177)
Observations	6217	6217	6217		11666	11666
**Panel E: CFPS 2018 wave**						
Conscientiousness	0.179*	0.306***	-0.011		0.723***	1.480***
	(0.106)	(0.094)	(0.115)		(0.265)	(0.344)
Extraversion	0.125	0.026	0.054		-0.197	0.108
	(0.090)	(0.079)	(0.097)		(0.176)	(0.219)
Agreeableness	-0.118	0.195***	-0.020		0.493***	0.469**
	(0.075)	(0.067)	(0.081)		(0.170)	(0.189)
Openness	0.033	0.067**	0.048		0.134	0.409***
	(0.040)	(0.034)	(0.041)		(0.099)	(0.105)
Neuroticism	0.035	-0.033	-0.109		0.104	0.085
	(0.087)	(0.076)	(0.095)		(0.159)	(0.192)
Observations	5381	5381	5381		10250	10250

*Notes*: All regressions control for cohort dummies, gender, marital status, minority, education level, health status, number of siblings, parents’ education level, whether parents are party members, urban dummies, and county fixed effects. Robust standard errors clustered at the county level are shown in parentheses.

*** significant at 1 percent level

** significant at 5 percent level

* significant at 10 percent level.

Second, I show the results of two-step Heckman model to alleviate sample selection bias. The first step consists of estimating a Probit model of whether the individual works, and the second equation is the occupational attainment equation. The results from the Heckman model in Table 5 are similar to the main findings.

Third, the evidence suggested that personalities are relatively stable among working-age adults whose adaptable thinking and behavioral patterns are established [56] and could be altered only in extreme cases, such as intensive psychotherapy and modifications to the brain [16, 19]. Thus I restrict the sample to those aged 30 to 60 and repeat the analysis. The coefficients change little in Panel C of Table 5 and the results are consistent with the main findings, suggesting the reverse causality concerns may be minor in the analysis.

The occupational status measures are not only available in CFPS 2010 survey but also CFPS 2016 and 2018 waves. I repeat the analysis with the occupational status variables from CFPS 2016 and 2018 waves respectively. The results in Panel D and Panel E in Table 5 indicate that although the coefficients change greatly due to different data waves, conscientiousness, agreeableness and openness are still the most important predictors of occupational attainment.

Finally, I use alternative indicators to measure one’s occupational status: one’s income level and self-rated social status. Based on the respondent’s answer to: “How much was your total income last year”, I generate income level variable. I also construct self-rated social status variable based on the respondent’s answer to “What is your social status in your local area”, with a higher value indicating higher social status. The results in Table 6 show that conscientiousness, agreeableness, and openness are positively associated with one’s income level. In contrast, neuroticism significantly reduces one’s income. The findings are consistent with the main regression results, which show that conscientiousness, agreeableness, and openness are beneficial to one’s occupational attainment.

**Table 6:** Robustness checks: Alternative measures for occupational status.

	Self-rated social status	Income level
Conscientiousness	0.237***	0.071*
	(0.029)	(0.097)
Extraversion	0.035*	-0.090
	(0.020)	(0.080)
Agreeableness	0.105***	0.182***
	(0.015)	(0.050)
Openness	0.047***	0.052*
	(0.009)	(0.027)
Neuroticism	-0.136***	-0.088*
	(0.017)	(0.048)
R^2^	0.135	0.241
Observations	16343	15910

*Notes*: All regressions control for cohort dummies, gender, marital status, minority, education level, health status, number of siblings, parents’ education level, whether parents are party members, urban dummies, and county fixed effects. Robust standard errors clustered at the province level are shown in parentheses.

*** significant at 1 percent level

** significant at 5 percent level

* significant at 10 percent level.

### Heterogeneity

In this subsection, I show different returns of personality traits according to different characteristics, such as gender, urban or rural areas.

Although the results in Table 7 suggest that there are no significant gender differences in personality traits returns to occupational prestige and socioeconomic status, all personality traits except extroversion have a stronger association with women’s occupational types. The findings are in line with the studies from developed countries [15, 34], which emphasize the gender differences in labor market returns of personality traits.

**Table 7:** Heterogeneity in terms of gender.

	Occupational types				
	(1)	(2)	(3)		
	Self-employed	Managers or professionals	Routine non-manual workers	Prestige scale	Socioeconomic status
Conscientiousness	0.451***	0.566***	0.347**	0.510	1.580***
	(0.172)	(0.159)	(0.160)	(0.317)	(0.347)
Extroversion	-0.240	0.011	0.016	0.021	-0.072
	(0.156)	(0.144)	(0.144)	(0.230)	(0.245)
Agreeableness	0.228*	0.360***	0.248**	0.333*	0.699***
	(0.138)	(0.124)	(0.125)	(0.175)	(0.200)
Openness	0.114*	0.163***	0.127**	0.172	0.430***
	(0.069)	(0.059)	(0.060)	(0.106)	(0.138)
Neuroticism	0.159	0.024	0.148	-0.401**	-0.114
	(0.140)	(0.132)	(0.131)	(0.163)	(0.187)
Conscientiousness * male	-0.363*	-0.144	-0.211	0.354	0.352
	(0.198)	(0.189)	(0.212)	(0.293)	(0.341)
Extroversion * male	0.288	0.107	-0.107	-0.067	0.032
	(0.186)	(0.180)	(0.201)	(0.248)	(0.310)
Agreeableness * male	-0.146	-0.152	-0.292*	0.246	-0.277
	(0.162)	(0.154)	(0.173)	(0.266)	(0.296)
Openness * male	-0.166**	-0.134*	-0.047	-0.012	-0.269
	(0.084)	(0.075)	(0.085)	(0.137)	(0.177)
Neuroticism * male	-0.319*	-0.223	-0.574***	0.218	-0.085
	(0.178)	(0.174)	(0.208)	(0.199)	(0.254)
Observations	4925	4925	4925	12451	12451

*Notes*: All regressions control for cohort dummies, marital status, minority, education level, health status, number of siblings, parents’ education level, whether parents are party members, urban dummies, and county fixed effects. Robust standard errors clustered at the county level are shown in parentheses.

*** significant at 1 percent level

** significant at 5 percent level

* significant at 10 percent level.

Specifically, the returns of conscientiousness, openness, and neuroticism to being self-employed are larger for women. In addition, the relationship between openness and being managers or professionals is also stronger for women. The results are consistent with the findings from Mueller and Plug, who examined the effects of personality on male-female earnings with a longitudinal survey of American high school graduates and suggested that the labor market appears to value conscientiousness and openness for women [35].

The association between agreeableness and neuroticism and being routine non-manual workers is also stronger for females. The results coincide with the findings, which suggested that agreeableness and neuroticism are two traits showing the largest gender differences [33].

Table 8 shows little consistency in the returns of personality traits to occupational attainment between urban and rural samples. On the one hand, the returns of conscientiousness and agreeableness to occupational prestige and socioeconomic status are larger for urban samples. On the other hand, the association between extraversion traits and occupational choices is pronounced for those from rural areas, where social networks are more beneficial in job searching and promoting in the labor market.

**Table 8:** Heterogeneity in terms of urban or rural community.

	Occupational types				
	(1)	(2)	(3)		
	Self-employed	Managers or professionals	Routine non-manual workers	Prestige scale	Socioeconomic status
Conscientiousness	0.358**	0.386*	0.564**	0.107	1.291***
	(0.172)	(0.197)	(0.244)	(0.206)	(0.300)
Extroversion	0.129	0.382**	0.312	0.127	-0.017
	(0.139)	(0.168)	(0.210)	(0.156)	(0.178)
Agreeableness	0.154	0.111	0.039	0.043	0.213
	(0.120)	(0.140)	(0.180)	(0.127)	(0.206)
Openness	0.025	0.171**	0.092	0.127	0.205
	(0.071)	(0.076)	(0.098)	(0.083)	(0.128)
Neuroticism	-0.043	-0.142	-0.086	-0.237**	-0.089
	(0.135)	(0.159)	(0.198)	(0.118)	(0.154)
Conscientiousness *urban	-0.246	0.108	-0.437	1.517***	1.261**
	(0.219)	(0.230)	(0.275)	(0.512)	(0.611)
Extroversion * urban	-0.327*	-0.454**	-0.473**	-0.362	-0.099
	(0.184)	(0.200)	(0.240)	(0.364)	(0.424)
Agreeableness * urban	-0.039	0.222	0.097	1.148***	0.919**
	(0.157)	(0.166)	(0.206)	(0.284)	(0.387)
Openness * urban	-0.030	-0.119	-0.011	0.088	0.190
	(0.087)	(0.087)	(0.108)	(0.169)	(0.190)
Neuroticism * urban	0.022	0.054	-0.001	-0.195	-0.217
	(0.175)	(0.187)	(0.225)	(0.310)	(0.358)
Observations	4925	4925	4925	12451	12451

*Notes*: All regressions control for cohort dummies, gender, marital status, minority, education level, health status, number of siblings, parents’ education level, whether parents are party members, urban dummies, and county fixed effects. Robust standard errors clustered at the county level are shown in parentheses.

*** significant at 1 percent level

** significant at 5 percent level

* significant at 10 percent level.

## Discussion

This paper estimates the relationship between personality traits measured by the “Big Five model” and occupational status using a nationally representative household survey from China. The results show that four of the five personality traits except extraversion are significantly associated with occupational status in terms of occupational choices, occupational prestige, and socioeconomic status. In particular, conscientiousness is the most important predictor among the five dimensions of personality traits. I also find that the returns of non-cognitive skills to occupational status are larger for females.

The findings highlight the important role of personality traits in China’s labor market. The results are in line with the studies from developed countries, which demonstrate that conscientiousness is the most stable and important trait for all occupations and occupational status [4]. The findings are also consistent with the study by Ham, Junankar and Wells [26], who found that all personality traits except extraversion are significantly associated with the probability of attaining a white collar occupation or a blue collar occupation. The results also coincide with the findings which suggest that openness to experience and agreeableness are related to job performance [32].

Personality traits are more malleable than cognitive ability over the life cycle and more sensitive to investments. Most of these skills are shaped in early life, therefore policy interventions in early childhood have a higher return, especially for disadvantaged children. With the economic development and application of artificial intelligence technology in the labor market, personality traits are playing an increasingly important role in occupational attainment. Individuals and parents should not only pay attention to cognitive competence such as mathematics and language skills but also foster personality traits. Individuals should develop competencies that are demanded by employers to get job opportunities and facilitate skills matching. Non-cognitive skills can also be developed in work environments, such as through job training with co-workers or supervisors. The company should pay attention to the importance of personality traits in the labor market and carry out skills training to improve one’s non-cognitive skills.

It should be noted that the personality traits measure is based on self-reported and I cannot fully account for possible confounding factors. Thus the estimates may be better interpreted as correlations, rather than causal effects of non-cognitive skills on occupational status. More work can be done on this issue if better-quality data is available.

### Endnotes

φ In the following robustness checks, I repeat the analysis with income level as outcome and the results are shown in Table 6.

κ Tables C and D in S1 File show that the coefficients of Big Five traits are stable to the inclusion of the other variables included in the equations.

## Supporting information

S1 File Appendix. (DOCX)

## References

[1] Heckman J, Stixrud N, Urzua S. The effects of cognitive and non-cognitive abilities on labor market outcomes and social behavior. Journal of Labor Economics. 2006; 24(3): 411–482.

[2] Heckman JJ, Kautz TD. Hard evidence on soft skills. NBER Working Paper Series. 2012; 19: 451–464. doi: 10.1016/j.labeco.2012.05.014 23559694 PMC3612993

[3] Cuesta MB, Budría S. Unemployment persistence: how important are non-cognitive skills? Journal of Behavioral and Experimental Economics. 2017; 69: 29–37.

[4] Lindqvist E, Vestman R. The labor market returns to cognitive and non-cognitive ability: evidence from the Swedish enlistment. American Economic Journal: Applied Economics. 2011; 3(1): 101–128.

[5] Mohanty MS. Effects of positive attitude and optimism on employment: evidence from the US data. Journal of Socio-Economics. 2010; 39(2): 258–270.

[6] Bühler D, Sharma R, Stein W. Occupational attainment and earnings in Southeast Asia: the role of non-cognitive skills. Labour Economics. 2020; 67.

[7] Adhitya D, Mulyaningsih T, Samudro BR. The role of cognitive and non-cognitive skills on labour market outcomes in Indonesia. Journal Ekonomi Malaysia. 2019; 53(1): 3–16.

[8] Cunningham W, Torrado M, Sarzosa M. Cognitive and non-cognitive skills for the Peruvian labor market: addressing measurement error through latent skills estimations. World Bank Policy Research Working Paper. 2016; 7550.

[9] Cheng H, Li T. The effects of personality traits on wages: empirical analysis based on the China Employer-Employee Survey (CEES) (in Chinese). Economic Research Journal. 2017; 2: 171–186.

[10] Campbell D, Ahmed I. The labour market in developing countries. Perspectives on Labour Economics for Development. 2012.

[11] Fields GS. Labor market analysis for developing countries. Labour Economics. 2011; 18: 16–22.

[12] Hilger A, Nordman CJ, Sarr LR. Cognitive and non-cognitive Skills, hiring channels, and wages in Bangladesh. IZA Discussion Paper. 2018; No. 11578.

[13] Brewer MB, Chen YR. Where (who) are collectives in collectivism? Toward conceptual clarification of individualism and collectivism. Psychological Review. 2007; 114(1): 133–151. doi: 10.1037/0033-295X.114.1.133 17227184

[14] DiTomaso N, Bian Y. The structure of labor markets in the US and China: social capital and guanxi. Management and Organization Review. 2018; 14(1): 5–36.

[15] Lesner RV. Labor market sorting on personality traits and the gender wage gap. Applied Economics Letters. 2020; 27(11): 940–944.

[16] Costa TJ, McCrae RR. Revised NEO personality inventory (NEO-PI-R) and NEO Five Factor inventory (NEO-FFI) professional manual. 1992; Technical Report, Odessa, FL, USA.

[17] Almlund M, Duckworth AL, Heckman J, Kautz T. Personality psychology and economics. IZA Discussion Paper. 2011; No.5500.

[18] Li T, Zhang W. Personality traits and stock investment: evidence from China (in Chinese). Economic Research Journal. 2015; 6:103–116.

[19] Gensowski M. Personality, IQ, and lifetime earnings. Labour Economics. 2018; 51: 170–183.

[20] Borghans L, Meijers F, Ter Weel B. The role of noncognitive skills in explaining cognitive test scores. Economic Inquiry. 2006; 46(1): 2–12.

[21] Rotter JB. Generalized expectancies for internal versus external control of reinforcement. Psychological Monographs: General and Applied. 1966; 80(1): 1–28. 5340840

[22] Rosenberg M. Rosenberg self-esteem scale (RSE). Acceptance and commitment therapy. Measures package. 1965; 61(52): 18.

[23] Eysenck HJ. Dimensions of personality: 16, 5 or 3? Criteria for a taxonomic paradigm. Personality and Individual Differences. 1991; 12(8): 773–790.

[24] Digman JM. Higher-order factors of the Big Five. Journal of Personality and Social Psychology. 1997; 73(6): 1246–1256. doi: 10.1037//0022-3514.73.6.1246 9418278

[25] Mincer J. Schooling, experience, and earnings. Human Behavior & Social Institutions. 1974; (2): 83–96.

[26] Ham R, Junankar PN, Wells R. Occupational choice: personality matters. IZA Discussion Papers. 2009; No. 4105.

[27] Antecol H, Cobb-Clark DA. The influence of non-cognitive skills on young people’s occupational choice. Retrieved from Claremont McKenna College website: http://www. claremontmckenna. edu/rdschool/summer_conference/CMC_5_28_Antecol. pdf, 2010.

[28] Becker GS. Nobel lecture: The economic way of looking at behavior. Journal of political economy. 1993; 101(3): 385–409.

[29] Blau PM, Gustad JW, Jessor R., et al. Occupational choice: a conceptual framework. ILR Review. 1956; 9(4): 531–543.

[30] Choi Y, Lee D. Psychological capital, big five traits, and employee outcomes. Journal of Managerial Psychology. 2014; 29(2): 122–140.

[31] Fletcher JM. The effects of personality traits on adult labor market outcomes: evidence from siblings. Journal of Economic Behavior & Organization. 2013; 89: 122–135.

[32] Rothmann S, Coetzer EP. The big five personality dimensions and job performance. Journal of Industrial Psychology. 2003; 29(1): 68–74.

[33] Bourchard TJ, Loehlin JC. Genes, evolution and personality. Behavior Genetics. 2001; 31: 243–273. doi: 10.1023/a:1012294324713 11699599

[34] Mueller G, Plug E. Estimating the effect of personality on male-female earnings. IZA Working Paper. 2006; No.1254.

[35] Grazier S, Sloane PJ. Accident risk, gender, family status and occupational choice in the UK. Labour Economics. 2008; 15(5): 938–957.

[36] Nyhus EK, Pons E. Personality and the gender wage gap. Applied Economics. 2012; 44(1): 105–118.

[37] Blázquez M, Herrarte A, Llorente-Heras R. Competencies, occupational status, and earnings among European university graduates. Economics of Education Review. 2018; 62:16–34.

[38] Smith TW. Personality as risk and relience in physical health. Current Directions in Psychological Science. 2006; 15(5): 227–231.

[39] Roberts BW, Kuncel NR, Shiner R, Caspi A, Goldberg LR. The power of personality: the comparative validity of personality traits, socioeconomic status, and cognitive ability for predicting important life outcomes. Perspectives on Psychological Science. 2007; 2(4): 313–345. doi: 10.1111/j.1745-6916.2007.00047.x 26151971 PMC4499872

[40] Levine R, Rubinstein Y. Smart and illicit: who becomes an entrepreneur and do they earn more? Quarterly Journal of Economics. 2017; 132(2): 963–1018.

[41] Carneiro P, Crawford C, Goodman A. The impact of early cognitive and non-cognitive skills on later outcomes. CEE Discussion Paper. 2007; No.0092.

[42] Liu ZH. The impact of non-cognitive skills on academic achievement: evidence on youth of China (in Chinese). Studies in Labor Economics. 2018; 6(6): 69–94.

[43] Luo S, Chen W, Jiang X. Non-cognitive ability and individual entrepreneurial choice: theoretical and empirical evidence (in Chinese). Studies in Labor Economics. 2020; 8(2): 101–119.

[44] Wang X, Yue Y, Zhu C. Non-cognitive abilities and entrepreneurship empirical analysis-based on “China’s Family Panel Studies” (in Chinese). Finance and Economics. 2018; 239(11): 13–21.

[45] Glewwe P, Huang Q, Park A. Cognitive skills, non-cognitive skills, and the employment and wages of young adults in rural China. 2011; 24–26.

[46] Zhu Z. Non-cognitive ability and entrepreneurial income of rural-urban migrants: facts and mechanisms (in Chinese). Population & Economics. 2021; 3:18–34.

[47] Xie Y, Hu J. An introduction to the China Family Panel Studies (CFPS). Chinese Sociological Review. 2014; 47(1): 3–29.

[48] Treiman DJ. Occupational presitge in comparative perspective. 1997; New York: Academic Press.

[49] Yang J, Ai D. Effect of the BIG FIVE personality traits on entrepreneurial probability: influence of China’s household registration system. Journal of Labor Research. 2019; 40: 487–503.

[50] Tsukahara I. The effect of family background on occupational choice. Labour. 2007; 21(4): 871–890.

[51] Sjögren A. Occupational choice and incentives: the role of family background[R]. IUI Working Paper, 2000.

[52] Knight J, Yueh L. The role of social capital in the labour market in China. Economics of Transition. 2008; 16(3), 389–414.

[53] Heineck G, Anger S. The returns to cognitive abilities and personality traits in Germany. Labor Economics. 2010; 17(3): 535–546.

[54] Heckman J. Sample selection bias as a specification error. Econometrica. 1979; 47(1):153–160.

[55] George JM, Zhou J. Understanding when bad moods foster creativity and good ones don’t: the role of context and clarity of feelings. Journal of Applied Psychology. 2002; 87(4): 687–697. doi: 10.1037/0021-9010.87.4.687 12184573

[56] Caspi A, Roberts BW. Personality development across the life course: the argument for change and continuity. Psychological Inquiry. 2001; 12(2): 49–66.

