# Supplementary material for: Personality traits and occupational status-evidence from China
Source: PLoS One. 2023 Apr 7;18(4):e0284050. doi: 10.1371/journal.pone.0284050 (PMC10081741; doi:10.1371/journal.pone.0284050)
Supplement: S1 File — (DOCX) [file pone.0284050.s001.docx]

**S1 file. Appendix**

**Table A. The Big Five Inventory and their facets**

| **Big Five Inventory** | **Facets/items** | **Survey Questions**  How much do you agree with each statement/ which describe the characteristics that may apply to you:  1 (strongly disagree)-5 (strongly agree) |
| --- | --- | --- |
| **Conscientiousness** | Order | Neatness/cleanliness of respondents’ clothing. |
|  |  | Cleanliness of respondent’s home. |
|  | Achievement striving | Feeling successful. |
|  |  | In today’s society, hard work is rewarded. |
|  | Deliberation | Respondent’s suspicion of the interview |
| **Extraversion** | Passion | Respondent’s courteousness |
|  | Gregariousness | Not lonely. |
|  | Positive emotions | Having fun in life. |
| **Openness** | Values | Having children to carry on the family name (reversed). |
| **Agreeableness** | Trust | Doubt or trust others. |
|  | Altruism | Not being disliked by others. |
|  | Obedience/compliance | Respondent’s level of cooperation during the interview. |
|  |  | Is it easy for you to get on well with others? |
| **Neuroticism** | Anxiety | Feel nervous (reversed). |
|  |  | Feel agitated or upset and cannot remain calm (reversed). |
|  | Depression | Feel depressed and cannot cheer up (reversed). |
|  | Vulnerability | Feel hopeless about the future (reversed). |
|  |  | Feel that everything is difficult (reversed). |
|  |  | Think life is meaningless (reversed). |

*Sources:* CFPS2010 and 2012 survey data.

**Table B. Descriptive statistics in terms of gender**

|  | **(1)** | **(2)** |  |
| --- | --- | --- | --- |
|  | **Male** | **Female** | **(1)-(2)** |
| **Panel A: Occupational status** |  |  |  |
| Socioeconomic status scores | 32.187 | 30.419 | 1.768*** |
| Prestige scores | 39.727 | 39.858 | -0.130 |
| **Panel B: Big Five Model** |  |  |  |
| Conscientiousness | 3.467 | 3.449 | 0.018** |
| Extraversion | 3.973 | 3.941 | 0.032** |
| Agreeableness | 3.849 | 3.815 | 0.035** |
| Openness | 1.967 | 1.979 | -0.012 |
| Neuroticism | 1.444 | 1.547 | -0.103*** |
| **Panel C: Individual characteristics** |  |  |  |
| Age | 41.839 | 40.625 | 1.214*** |
| Male (%) | 1 | 0 | - |
| Married (%) | 88.000 | 91.387 | -3.387*** |
| Ethnic minority (%) | 8.242 | 8.494 | -0.252 |
| *Education level* |  |  |  |
| Primary school (%) | 38.530 | 52.863 | -14.332*** |
| Middle school (%) | 36.788 | 27.876 | 8.912*** |
| High school or above (%) | 24.682 | 19.262 | 5.420*** |
| Self-rated health status | 4.376 | 4.186 | 0.190*** |
| Urban hukou (%) | 24.561 | 20.441 | 4.120*** |
| **Panel D: Household characteristics** |  |  |  |
| Number of siblings | 2.925 | 3.065 | -0.140*** |
| *Father’s education level* |  |  |  |
| Primary school (%) | 76.227 | 74.620 | 1.608** |
| Middle school (%) | 14.273 | 15.998 | -1.725** |
| High school or above (%) | 9.500 | 9.383 | 0.117 |
| *Mother’s education level* |  |  |  |
| Primary school (%) | 88.879 | 87.489 | 1.389** |
| Middle school (%) | 7.848 | 8.631 | -0.783* |
| High school or above (%) | 3.273 | 3.880 | -0.610** |
| Father is party member | 15.864 | 15.792 | 0.071 |
| Mother is party member | 2.136 | 2.427 | -0.291 |
| Urban community (%) | 40.136 | 37.549 | 2.588** |

*Sources:* CFPS 2010 and 2012 survey data.

**Table C. Non-cognitive skills and occupational types**

|  | (1) | | | (2) | | | (3) | | |
| --- | --- | --- | --- | --- | --- | --- | --- | --- | --- |
|  | Self-employed | Managers or professionals | Routine  non-manual workers | Self-employed | Managers or professionals | Routine  non-manual workers | Self-employed | Managers or professionals | Routine  non-manual workers |
| Conscientiousness | 0.219** | 0.504*** | 0.234* | 0.227** | 0.487*** | 0.230* | 0.226** | 0.480*** | 0.241** |
|  | (0.111) | (0.108) | (0.122) | (0.111) | (0.108) | (0.122) | (0.111) | (0.108) | (0.122) |
| Extraversion | -0.047 | 0.064 | -0.025 | -0.052 | 0.067 | -0.018 | -0.057 | 0.069 | -0.017 |
|  | (0.093) | (0.093) | (0.104) | (0.093) | (0.093) | (0.104) | (0.093) | (0.093) | (0.104) |
| Agreeableness | 0.124 | 0.266*** | 0.107 | 0.125 | 0.257*** | 0.101 | 0.132* | 0.260*** | 0.098 |
|  | (0.079) | (0.078) | (0.089) | (0.079) | (0.078) | (0.089) | (0.079) | (0.078) | (0.089) |
| Openness | 0.008 | 0.080** | 0.088** | 0.008 | 0.078** | 0.088** | 0.005 | 0.081** | 0.088** |
|  | (0.042) | (0.038) | (0.043) | (0.042) | (0.038) | (0.043) | (0.042) | (0.038) | (0.043) |
| Neuroticism | -0.031 | -0.106 | -0.093 | -0.029 | -0.110 | -0.091 | -0.028 | -0.109 | -0.090 |
|  | (0.090) | (0.089) | (0.101) | (0.090) | (0.089) | (0.101) | (0.090) | (0.089) | (0.101) |
|  |  |  |  |  |  |  |  |  |  |
| Individual characteristics | √ | √ | √ | √ | √ | √ | √ | √ | √ |
| Family characteristics |  |  |  | √ | √ | √ | √ | √ | √ |
| Community characteristics |  |  |  |  |  |  | √ | √ | √ |
| Observations | 4925 | 4925 | 4925 | 4925 | 4925 | 4925 | 4925 | 4925 | 4925 |

*Notes:* Robust standard errors clustered at the county level are shown in parentheses. *** significant at 1 percent level, ** significant at 5 percent level, * significant at 10 percent level.

**Table D. Non-cognitive skills and occupational prestige and socioeconomic status**

|  | (1) | | (2) | | (3) | |
| --- | --- | --- | --- | --- | --- | --- |
|  | Prestige scale | Socioeconomic status | Prestige scale | Socioeconomic status | Prestige scale | Socioeconomic status |
| Conscientiousness | 0.750*** | 1.783*** | 0.714*** | 1.728*** | 0.689** | 1.772*** |
|  | (0.263) | (0.334) | (0.261) | (0.332) | (0.267) | (0.318) |
| Extraversion | -0.021 | -0.028 | -0.027 | -0.033 | -0.012 | -0.061 |
|  | (0.175) | (0.193) | (0.175) | (0.194) | (0.176) | (0.194) |
| Agreeableness | 0.466*** | 0.583*** | 0.456*** | 0.566*** | 0.463*** | 0.554*** |
|  | (0.124) | (0.175) | (0.123) | (0.173) | (0.123) | (0.174) |
| Openness | 0.163* | 0.305*** | 0.158* | 0.299*** | 0.164* | 0.287*** |
|  | (0.084) | (0.108) | (0.084) | (0.108) | (0.083) | (0.109) |
| Neuroticism | -0.307** | -0.120 | -0.316** | -0.132 | -0.301** | -0.158 |
|  | (0.128) | (0.156) | (0.129) | (0.157) | (0.128) | (0.159) |
|  |  |  |  |  |  |  |
| Individual characteristics | √ | √ | √ | √ | √ | √ |
| Family characteristics |  |  | √ | √ | √ | √ |
| Community characteristics |  |  |  |  | √ | √ |
|  |  |  |  |  |  |  |
| R^2^ | 0.145 | 0.432 | 0.148 | 0.436 | 0.151 | 0.439 |
| Observations | 12451 | 12451 | 12451 | 12451 | 12451 | 12451 |

*Notes:* Robust standard errors clustered at the county level are shown in parentheses. *** significant at 1 percent level, ** significant at 5 percent level, * significant at 10 percent level.
